# Inhibition of Influenza A Virus Infection by Fucoidan Targeting Viral Neuraminidase and Cellular EGFR Pathway

**DOI:** 10.1038/srep40760

**Published:** 2017-01-17

**Authors:** Wei Wang, Jiandong Wu, Xiaoshuang Zhang, Cui Hao, Xiaoliang Zhao, Guangling Jiao, Xindi Shan, Wenjing Tai, Guangli Yu

**Affiliations:** 1 Key Laboratory of Marine Drugs, Ministry of Education, Ocean University of China, Qingdao, 266003, P.R. China; 2 Shandong Provincial Key Laboratory of Glycoscience and Glycotechnology, Ocean University of China, Qingdao, 266003, P. R. China; 3 Institute of Cerebrovascular Diseases, Affiliated Hospital of Qingdao University Medical College, Qingdao, 266003, P. R. China; 4 Laboratory for Marine Drugs and Bioproducts of Qingdao National Laboratory for Marine Science and Technology, Qingdao, 266237, P. R. China

## Abstract

Development of novel anti-influenza A virus (IAV) drugs with high efficiency and low toxicity is critical for preparedness against influenza outbreaks. Herein, we investigated the anti-IAV activities and mechanisms of fucoidan *in vitro* and *in vivo*. The results showed that a fucoidan KW derived from brown algae *Kjellmaniella crassifolia* effectively blocked IAV infection *in vitro* with low toxicity. KW possessed broad anti-IAV spectrum and low tendency of induction of viral resistance, superior to the anti-IAV drug amantadine. KW was capable of inactivating virus particles before infection and blocked some stages after adsorption. KW could bind to viral neuraminidase (NA) and inhibit the activity of NA to block the release of IAV. KW also interfered with the activation of EGFR, PKCα, NF-κB, and Akt, and inhibited both IAV endocytosis and EGFR internalization in IAV-infected cells, suggesting that KW may also inhibit cellular EGFR pathway. Moreover, intranasal administration of KW markedly improved survival and decreased viral titers in IAV-infected mice. Therefore, fucoidan KW has the potential to be developed into a novel nasal drop or spray for prevention and treatment of influenza in the future.

Influenza A virus (IAV) is a most formidable pathogen, which has been the cause of at least three pandemics in the last century. The most severe IAV pandemic caused more than 40 million deaths in the world during 1918–1919^1^,^2^. In late April 2009, a novel influenza A (H1N1) virus caused a pandemic within a short period of time, which attracted great attention all over the world. Current anti-IAV drugs are directed against the viral M2 protein (adamantane and rimantadine) and neuraminidase (zanamivir and oseltamivir)^3^,^4^. Despite these successes, drug resistance, toxicity, and cost remain unresolved issues in the fight against IAV infection^5^,^6^,^7^. Hence, the development of novel anti-IAV agents that could be used alone or in combination with existing antiviral drugs is of high importance.

Influenza A virus can enter host cells by clathrin-mediated endocytosis or macropinocytosis after the virus binds to sialic acid residues *via* the viral hemagglutinin (HA)^8^. Eierhoff *et al*. reported that epidermal growth factor receptor (EGFR) can promote uptake of IAV into host cells, and the PI3K/Akt signaling pathway which can be activated by EGFR can also enhance IAV uptake^8^. Moreover, the viral neuraminidase (NA) protein was reported to be able to promote IAV entry into target cells during the initial stage of virus infection, in addition to promote the release process of progeny virus from host cells^9^. Thus, inhibitors of cellular EGFR pathway and viral NA protein may be used alone or in combination with other drugs to block both the invasion and release process of IAV.

Fucoidan, a sulfated polysaccharide found mainly in brown algae, was reported to possess a variety of biological activities, including anti-coagulant^10^, anti-viral^11^,^12^,^13^,^14^, anti-tumor^15^, and anti-inflammatory effects^16^. The functional properties of fucoidan make it an attractive target for the development of biomaterials and drugs^17^. Hayashi *et al*. reported that a fucoidan isolated from *Undaria pinnatifida* possessed anti-IAV activities in mice with normal and compromised immunity^18^. Synytsya and co-workers reported that the *Mekabu* fucoidan could inhibit avian IAV replication through enhancing immune system in mice^19^. Moreover, fucoidans could be used as vaccine adjuvants to activate spleen cells and enhance antigen-specific antibody production in mice^20^. Therefore, fucoidans have the potential to be developed into novel anti-IAV agents in the future.

To further correlate the potential anti-IAV applications of fucoidan with its underlying molecular mechanisms, the anti-IAV actions and mechanisms of fucoidan were investigated *in vitro* and *in vivo* in this study. The results showed that the fucoidan KW derived from brown algae *Kjellmaniella crassifolia* possessed broad anti-IAV spectrum and low tendency of induction of viral resistance. KW may possibly block IAV invasion and release process by targeting viral neuraminidase and cellular EGFR pathway.

## Results

### Inhibition of influenza A virus multiplication *in vitro* by fucoidan polysaccharides

**Figure 1:**
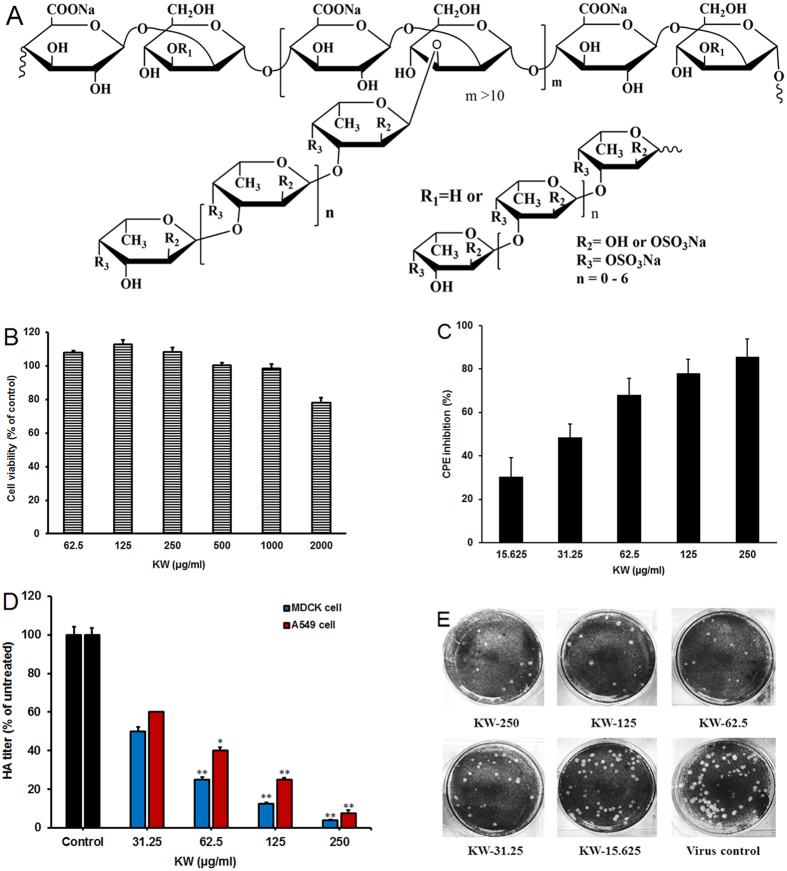
Fucoidan KW inhibited replication of IAV *in vitro* with low toxicity. (**A**) Schematic diagram of the chemical structure of fucoidan KW. (**B**) MDCK cells were exposed to different concentrations of KW in triplicate, and incubated at 37 °C for 48 h. Then the cell viability was evaluated by MTT assay. The results were presented as a percentage of control group. Values are means ± S.D. (n = 3). (**C**) PR8 virus (MOI = 0.1) infected MDCK cells were treated with KW at the indicated concentrations after removal of virus inoculums. The antiviral activity was determined by CPE inhibition assay at 48 h p.i. Results are expressed as percent of inhibition in drug-treated cultures compared with untreated. Values are means ± S.D. (n = 5). (**D**) IAV (MOI = 0.1) infected MDCK and A549 cells were treated with KW at the indicated concentrations after removal of the virus inoculum. The antiviral activity was determined by hemagglutination (HA) assay at 48 h p.i. Mean percentage HA titers were calculated as a percentage of HA titers from untreated control group. Values are means ± S.D. (n = 4). Significance: *p < 0.05, **p < 0.01 vs. virus control group. (**E**) Approximately 50–100 PFU/well of PR8 was pre-incubated with different concentrations of KW for 60 min at 37 °C before infection. Then the virus-KW mixture was transferred to confluent cell monolayers in 6-well plates, incubated at 37 °C for 1 h and subjected to plaque assay.

The fucoidan KW was extracted from brown algae *Kjellmaniella crassifolia* following the methods described previously^21^. The average molecular weight of KW determined by gel filtration chromatography was about 536 kDa (Table 1). The sulfate content of KW was 30.1% as determined by the method of Dodgson and Price^22^ (Table 1), and the purity of KW was more than 98% as determined by HPLC. The structure of KW was determined by nuclear magnetic resonance spectroscopy (NMR) and electrospray ionization mass spectrometry (ESI-MS) analysis, which showed that KW is a 3-linked 2,4-*O*-disulfated fucooligosaccharide branched glucuronomannan (Fig. 1A)^23^.

The cytotoxicity of KW was firstly evaluated by MTT assay^24^. The results showed that KW exhibited no significant cytotoxicity at the concentrations from 62.5 to 2000 μg/ml (Fig. 1B). KW showed some cytotoxicity to MDCK cells at 2000 μg/ml but without statistical significance. The maximum non-toxic concentration was about 1000 μg/mL (Fig. 1B). Moreover, the CC_50_ (50% Cytotoxicity Concentration) value for KW was about 2752.6 μg/ml (Table 1).

KW was then assayed for its ability to inhibit IAV multiplication *in vitro* using CPE inhibition assay and hemagglutination (HA) assay^25^,^26^. MDCK cells were initially infected with influenza virus (A/Puerto Rico/8/34 (H1N1); PR8) (MOI = 0.1), and then treated with KW at the indicated concentrations after removal of the virus inoculum. At 48 hours post infection (p.i.), the viral titers in the culture media were determined by HA assay, and cell viability was measured by CPE inhibition assay. As shown in Fig. 1C,D, KW significantly reduced the virus HA titer and promote cell viability when used at the concentration >62.5 μg/mL (*p* < 0.01). The 50% inhibitory concentration (IC_50_ value) of KW for CPE inhibition was about 34.4 μg/mL, and the selectivity index (CC_50_/IC_50_) for KW was approximately 80.0, which was superior to that of ribavirin (SI = 31.0) and slightly less than that of oseltamivir carboxylate (SI = 88.1) (Table 1). Moreover, the HA assay was also performed in human lung epithelial cells (A549 cells) to explore whether the inhibition of IAV by KW was cell-specific or not. As shown in Fig. 1D, viral replication in A549 cells was also dose-dependently inhibited by KW and KW significantly reduced the virus HA titer when used at the concentration >62.5 μg/mL ((*p* < 0.05).

To explore whether KW had direct inhibition actions on viral particles, the plaque reduction assay was performed as described previously^27^. In brief, PR8 virus (50–100 PFU/well) was pre-incubated with or without KW for 60 min at 37 °C before infection. Then the virus-KW mixture was transferred to confluent cell monolayers in 6-well plates, incubated at 37 °C for 1 h and subjected to plaque assay. As shown in Fig. 1E, pre-incubation of PR8 with KW at the concentrations of 31.25–250 μg/ml markedly reduced the number of plaques and protected MDCK cells, suggesting that KW may be able to inactivate viral particles directly.

### KW possesses broad anti-IAV spectrum and low tendency of induction of viral resistance.

#### Effects of KW on a single cycle of virus replication

**Figure 2:**
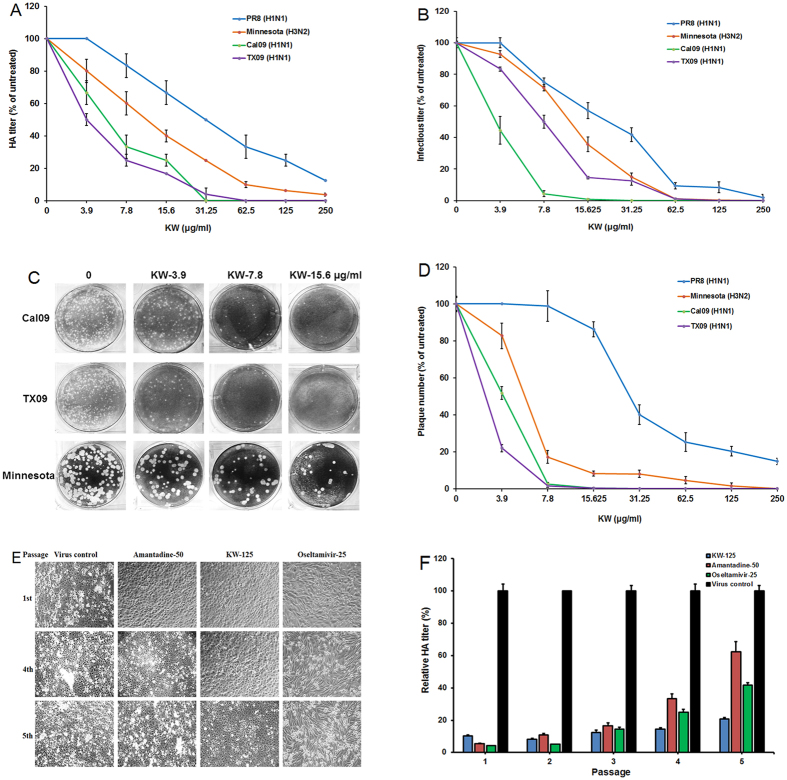
KW possesses broad-spectrum anti-IAV activities and low tendency of induction of viral resistance. (**A**) HA titers and (**B**) infectious virus titers from single-cycle high-moi assays performed on MDCK cells infected with PR8, Minnesota, Cal09 and TX09 and treated with the indicated concentrations of KW. Mean percentage HA titers or infectious virus titers were calculated as a percentage of HA or infectious virus titers, respectively, from untreated cells for each drug treatment condition in an experiment. Values are means ± S.D. (n = 4). (**C**) Approximately 50–100 PFU/well of Cal09, TX09 or Minnesota virus were pre-incubated with KW (0, 3.9, 7.8, 15.6 μg/ml) for 1 h at 37 °C before infection, respectively. Then the virus-KW mixture was transferred to MDCK cells, incubated at 37 °C for 1 h and subjected to plaque reduction assay. (**D**) Plaque number from plaque reduction assays performed on MDCK cells infected with the four viruses and treated with the indicated concentrations of KW. Values are means ± S.D. (n = 4). (**E**) Microscopy observations of CPE at the 1st, 4th and 5th passage of a multi-passaging experiment treated by either KW (125 μg/ml), amantadine (50 μg/ml) or Oseltamivir (25 μg/ml). (**F**) Quantitative analysis of the relative yield of progeny virus by HA assay at each round of total five rounds of propagation. PR8 (MOI = 0.1) infected MDCK cells were treated with KW, amantadine or Oseltamivir. At 24 h p.i., the cell supernatants were collected and employed for infection in the next round of investigation. Virus yields of mock-treated cells were arbitrarily set as 100%.

Since the PR8 virus was isolated several decades ago, we were interested whether KW possesses antiviral activities against the current pandemic human and swine strains. Thus, the inhibition of KW on the virus yields from MDCK cells infected with PR8 (H1N1), Cal09 (A/California/04/2009; H1N1), Minnesota (A/swine/Minnesota/02719/2009; H3N2), and TX09 (A/Texas/15/2009; H1N1), at high moi (≈3.0 PFU/cell) were examined by HA assay and plaque assay. Briefly, MDCK cells were infected with KW-pretreated IAV and treated with KW after the infection period. At 24 h p.i., the HA titers and infectious virus titers of cell culture supernatants were determined. As shown in Fig. 2A,B, for all four viruses tested, the reduction of virus yields measured by both HA titer and infectious virus titer with increasing concentrations of KW were in a dose dependent manner. The IC_50_ values obtained for KW inhibition of PR8 virus were higher than that of Minnesota (H3N2) and the pandemic H1N1 virus (Cal09 and TX09) (Table 2). At the concentration of 31.25 μg/ml, the HA titers were reduced to about 50% of the untreated control for PR8, 25% of the control for Minnesota, 5% of the control for TX09 and 0% of the control for Cal09 (Fig. 2A).

Moreover, for each virus tested, the reduction in infectious virus titer corresponded to the reduction in HA titer with increasing concentrations of KW (Fig. 2B). The IC_50_ values obtained for KW inhibition of Cal09 (3.5 ± 0.9 μg/ml) and TX09 (8.4 ± 1.3 μg/ml) were lower than that of PR8 (30.7 ± 2.9 μg/ml) and Minnesota (21.4 ± 1.5 μg/ml) (Table 2). At the concentration of 31.25 μg/ml, the infectious virus titer was reduced to about 40% of the untreated control for PR8, 15% of the control for Minnesota, 12.5% of the control for TX09 and 0.2% of the control for Cal09 (Fig. 2B). Therefore, the pandemic H1N1 virus (Cal09) may be the virus that was most susceptible to KW treatment.

#### Effects of KW over multiple cycles of infection

The inhibition effects of KW on IAV infection was also examined over multiple cycles of infection using plaque reduction assay^27^. Briefly, MDCK cells were infected with KW pretreated virus (PR8, Cal09, TX09 or Minnesota) at an moi of 0.003 pfu for 1 h at 37 °C, and then subjected to plaque assay. As shown in Fig. 2C, KW significantly inhibited the plaque formation in Cal09, TX09 and Minnesota infected cells when used at the concentration >3.9 μg/ml. For PR8 virus, the IC_50_ value for plaque number reduction was about 30.5 ± 3.7 μg/ml (Table 2). However, in Minnesota-infected cells an obvious inhibition of KW on plaque formation was observed and the IC_50_ value for Minnesota was only 6.3 ± 0.1 μg/ml, largely superior to that for PR8 virus (Fig. 2D and Table 2). KW showed more significant inhibition on plaque formation in Cal09 and TX09 infected cells, with IC_50_ values of 3.8 ± 0.2 and 2.9 ± 0.1 μg/ml, respectively (Table 2). Thus, KW could inhibit both H1N1 and H3N2 virus multiplication *in vitro*.

#### KW possesses low tendency of induction of viral resistance

To explore whether KW induces the IAV to produce drug resistance, a multi-passage experiment based on CPE inhibition assay and HA assay was performed^27^. Based on the results of CPE inhibition assay, the IC_50_ values of KW, amantadine and Oseltamivir against PR8 virus are about 34.4, 23.8 and 13.1 μg/ml, respectively (Table 1). Thus, 125 μg/ml of KW, 50 μg/ml of amantadine, and 25 μg/ml of Oseltamivir (~2–4 times of IC_50_) were used in the multi-passaging experiment. As shown in Fig. 2E,F, the results showed that a remarkable viral resistance is induced by amantadine or Oseltamivir, suggesting that a low-level replication is allowed which gives the IAV a chance to adapt to the selective pressure of amantadine or Oseltamivir (Fig. 2E). However, KW could still markedly reduce the virus titer in culture media and promote cell viability after the fourth and fifth passage, suggesting that KW was still efficient in inhibiting PR8 propagation (Fig. 2E,F). Therefore, KW possesses broad-spectrum anti-IAV activities and low risk of inducing drug resistance.

#### Influence of different treatment conditions of KW on IAV infection

**Figure 3:**
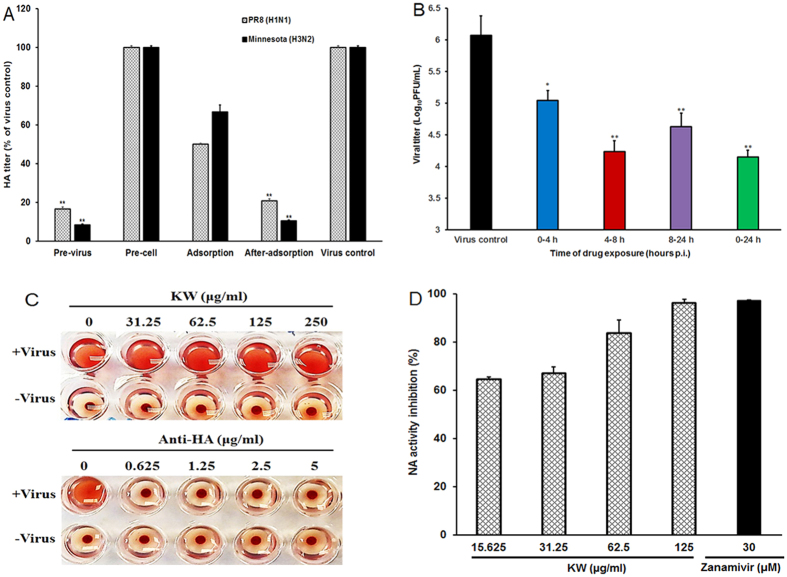
Influence of different treatment conditions of fucoidan on IAV infection. (**A**) MDCK cells were infected with Minnesota (H3N2) or PR8 (MOI = 0.1) under four different treatment conditions. (i) Pretreatment of virus: IAV was pretreated with 250 μg/mL of KW at 37 °C for 1 h before infection. (ii) Pretreatment of cells: MDCK cells were pretreated with 250 μg/mL of KW before infection. (iii) Adsorption: cells were infected in media containing 250 μg/mL of KW and, after 1 h adsorption at 37 °C, were overlaid with compound-free media. (iv) After adsorption: after removed unabsorbed virus the infecting media containing 250 μg/mL of KW were added to cells. At 24 h p.i., the antiviral activity was determined by HA assay. Values are means ± S.D. (n = 3). Significance: **p < 0.01 vs. virus control group. (**B**) PR8 (MOI = 0.1) infected MDCK cells were treated with 250 μg/mL of KW for the specified time period, and then the media were removed and cells were overlaid with compound-free media. Then at 24 h p.i., the cell supernatants were collected and the virus yields were determined by plaque assay. Values are means ± S.D. (n = 3). Significance: *p < 0.05 vs. virus control group. (**C**) The inhibition effects of KW and anti-HA antibody on IAV-induced aggregation of chicken erythrocytes were evaluated by hemagglutination inhibition (HI) assay. (**D**) Inactivated PR8 virus was incubated with indicated concentrations of KW or Zanamivir (30 μM), and the NA enzymatic activity was determined by a fluorescent assay. Values are means ± S.D. (n = 4).

Various time-points were assessed to determine the stage(s) at which KW exerted its inhibitory effects in *vitro*. In brief, MDCK cells were infected with Minnesota (H3N2) or PR8 (MOI = 0.1) under four different treatment conditions: pre-treatment of viruses, pre-treatment of cells, during virus adsorption, or after adsorption. At 24 h p.i., the antiviral activity was determined by HA assay^26^,^27^. As shown in Fig. 3A, pretreatment of Minnesota or PR8 with 250 μg/ml KW for 1 h before infection significantly inhibited the virus HA titers compared to that in virus control group (*p* < 0.01), suggesting that KW may have direct interaction with IAV particles. However, either the addition of KW during virus adsorption or pretreatment of cells did not significantly decrease the virus titers (Fig. 3A), which suggested that KW may not interact with MDCK cells directly. Interestingly, post-treatment of cells with KW after virus adsorption also significantly reduced the virus titers as compared to that in virus control group (*p* < 0.01) (Fig. 3A). Thus, KW may be able to inactivate virus particles directly and block some stages after virus adsorption.

Moreover, another time course study was also performed to explore which viral stage after adsorption is inhibited by KW as described previously^28^. Briefly, IAV (MOI = 1.0)-infected MDCK cells were treated with 250 μg/mL of KW for different time intervals, then the virus yields at 24 h p.i. were evaluated by plaque assay. The results showed that KW treatment for the first 4 h (0–4 h p.i.) after adsorption resulted in a significant reduction of virus titer (about 10-fold) (*p* < 0.05) (Fig. 3B), which suggested that KW may be able to inhibit IAV entry. However, greater inhibition was noted (about 100-fold) when KW was added 4 h after infection (4–8 h p.i.) (*p* < 0.01), and it was almost as effective as that in the group with KW treatment during 0–24 h p.i (Fig. 3B), suggesting that KW may also be able to inhibit newly released IAV from infecting cells.

Since KW may interact directly with virus particles, we then explored whether KW had interaction with virus surface HA protein by using the hemagglutination inhibition (HI) assay. The results showed that the anti-HA antibodies significantly inhibited the PR8 virus-induced aggregation of chicken erythrocytes at the concentrations of 0.625–5 μg/mL (Fig. 3C), which suggested that the anti-HA antibody can obstruct the virus attachment to red blood cells through binding to HA. However, KW could not inhibit virus-induced aggregation of chicken erythrocytes even at a concentration of 250 μg/ml (Fig. 3C), suggesting that KW may have no direct interaction with viral HA protein.

We next asked if the decreased virus titer was due to direct inhibition of viral NA activity by performing NA inhibition assay^29^,^30^. To address this question, we tested the inhibition effects of KW on the NA activity and compared it to the effect of Zanamivir, a well-known NA inhibitor^29^,^30^. As shown in Fig. 3D, KW inhibited the NA activity of the PR8 virus at low IC_50_ value (8.8 μg/ml), and the inhibition effect of KW on NA activity was in a dose-dependent manner at the concentrations of 15.625–125 μg/mL, suggesting that KW may directly bind to viral NA protein to inhibit IAV infection.

#### KW inhibits the neuraminidase activity of IAV to block virus release

**Figure 4:**
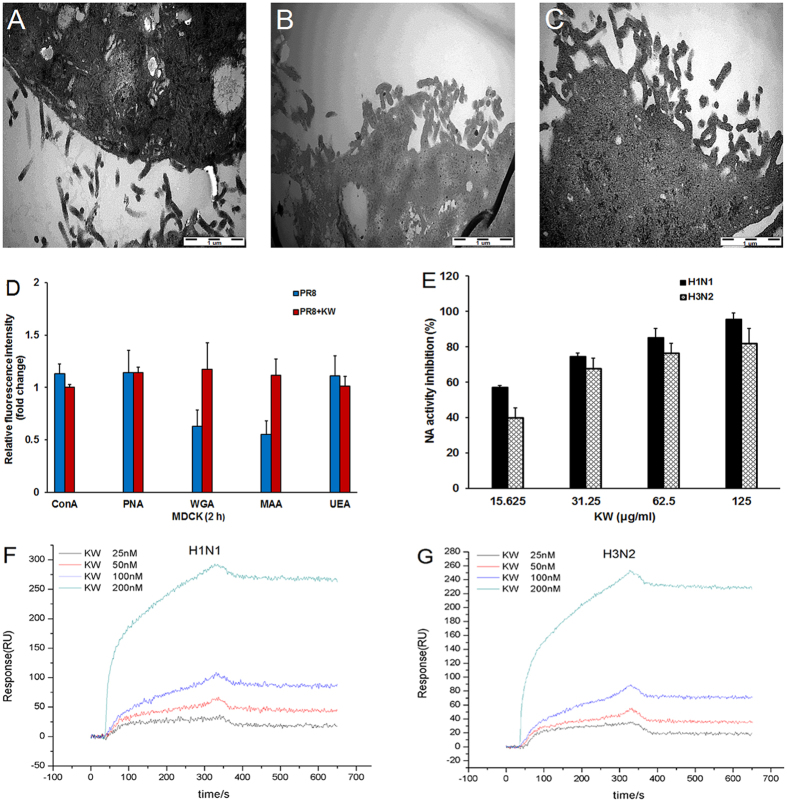
KW inhibited the neuraminidase activity of IAV to block virus release. (**A–C**) MDCK cells were infected with PR8 virus and exposed to PBS, Zanamivir or KW, then processed for electron microscopy at 24 h p.i. (**A**) MDCK cells infected with PR8 virus in the presence of PBS only. (**B**) MDCK cells infected with PR8 virus in the presence of Zanamivir (30 μM). (**C**) MDCK cells infected with PR8 in the presence of KW (250 μg/ml). Scale bar represents 1 μm. (**D**) IAV infected MDCK cells were treated with or without KW at 250 μg/ml for 2 h, and then different kinds of FITC-labeled lectins (20 μg/ml) were added and incubated at 37 °C for 30 min. The fluorescence intensity (FI) of each sample was measured by fluorescence microplate reader. The FI for non-infected control group was assigned values of 1.0. Values are means ± S.D. (n = 3). (**E**) Two different kinds of recombinant NA proteins (H1N1 or H3N2 subtype) were incubated with indicated concentrations of KW and the NA enzymatic activity was determined by a fluorescent assay. The fluorescence intensity was measured using a SpectraMax M5 plate reader with excitation and emission wavelengths of 360 and 440 nm, respectively. Values are means ± S.D. (n = 3). (**F** and **G**) The NA proteins of H1N1 subtype (**F**) or H3N2 subtype (**G**) were firstly immobilized onto the surface of a carboxymethylated dextran sensor chip (CM5). To assess real-time binding of KW to the NA proteins on CM5 chips, KW at given concentrations (200, 100, 50, 25 nM) was flowed over the biosensor chip surface. The sensorgram for all binding interactions were recorded in real time and were analyzed after subtracting the sensorgram from the blank channel. Then, the changes in mass due to the binding response were recorded as resonance units (RU).

Since KW may interact with NA protein to inhibit its activity, we then explored whether KW could block the release of IAV. Briefly, the PR8 virus infected MDCK cells were treated with KW or Zanamivir for 24 h and then examined by electron microscopy. As shown in Fig. 4A–C, compared to the untreated PR8-infected cells (Fig. 4A), Zanamivir treatment obviously inhibited the release of IAV and induced the aggregation of IAV particles (Fig. 4B). Similar to the phenomenon in Zanamivir-treated cells, KW treatment also induced viral aggregation on the cell surface at 24 h p.i. (Fig. 4C), suggesting that KW may interfere with the release of IAV, just like other neuraminidase inhibitors^29^. Therefore, KW may inhibit IAV infection through inhibiting the enzymatic activity of NA to block virus release.

To explore the influence of KW on the hydrolysis of sialic acids on cell surface by NA, FITC-labeled lectins were used to determine the amount of cell surface glycans. As shown in Fig. 4D, at 2 h p.i., the α-D-mannose-containing N-glycans (recognized by ConA), Galβ1-3GalNAcα-containing O-glycans (recognized by PNA), and α-L-fucose-containing N, O-glycans (recognized by UEA-I) had little change compared to that in non-infected cells. However, the Neu5Ac (sialic acid)-containing glycans, recognized by WGA, apparently decreased compared to that in non-infected cells (≈0.6-fold), which may be due to the hydrolysis of sialic acids by NA (Fig. 4D). In contrast, after treatment with KW for 2 h, the Neu5Ac-containing glycans almost restored to the same level as that in non-infected cells (≈1.1-fold) (Fig. 4D). Moreover, the 2′3-linked sialic acids highly expressed on the surface of MDCK cells, recognized by MAAI, displayed the same change tendency as the total sialic acids recognized by WGA (Fig. 4D), which suggested that KW truly inhibited the hydrolysis of sialic acid residues on cell surface by IAV.

To further explore whether the inhibition of NA activity by KW was subtype-specific or not, the NA inhibition assay was performed with two kinds of recombinant NA proteins (A/California/04/2009 (H1N1) and A/Babol/36/2005 (H3N2)). As shown in Fig. 4E, KW markedly inhibited the enzymatic activities of these two NA proteins at the concentration >31.25 μg/ml (all more than 70%), and the inhibition effects of KW were all in a dose-dependent manner at the concentrations of 15.625–125 μg/ml. The inhibition effects of KW on Cal09 NA protein was a little higher than that on H3N2 NA protein (Fig. 4E).

Moreover, the interaction between KW and NA protein was further evaluated by using SPR assay^31^,^32^. Briefly, with NA proteins being immobilized on the chip, KW at the concentrations of 25–200 nM (about 12.5–100 μg/ml) was flowed over the biosensor chip surface, respectively. Data revealed a marked binding of KW to Cal09 (H1N1) NA in a concentration-dependent manner with a KD equivalent to about 1.22E-8 M (12.2 nM), implicating a high affinity of KW for Cal09 NA (Fig. 4F). Thus, pretreatment of IAV with KW before infection may allow KW to fully bind NA and form a stable KW-NA complex. In addition, KW also bound to H3N2 NA in a dose-dependent manner with a KD equivalent to about 1.81E-8 M (18.1 nM), suggesting that KW could bind to the NA proteins of two different subtypes (H1N1 and H3N2) specifically (Fig. 4G). In contrast, KW weakly bound to HA protein with a much higher KD value (about 300 μM) (data not shown). Therefore, KW may directly bind to NA protein and inhibit its activity to block the invasion and release of IAV.

#### KW reduced IAV endocytosis through inhibition of EGFR pathway

**Figure 5:**
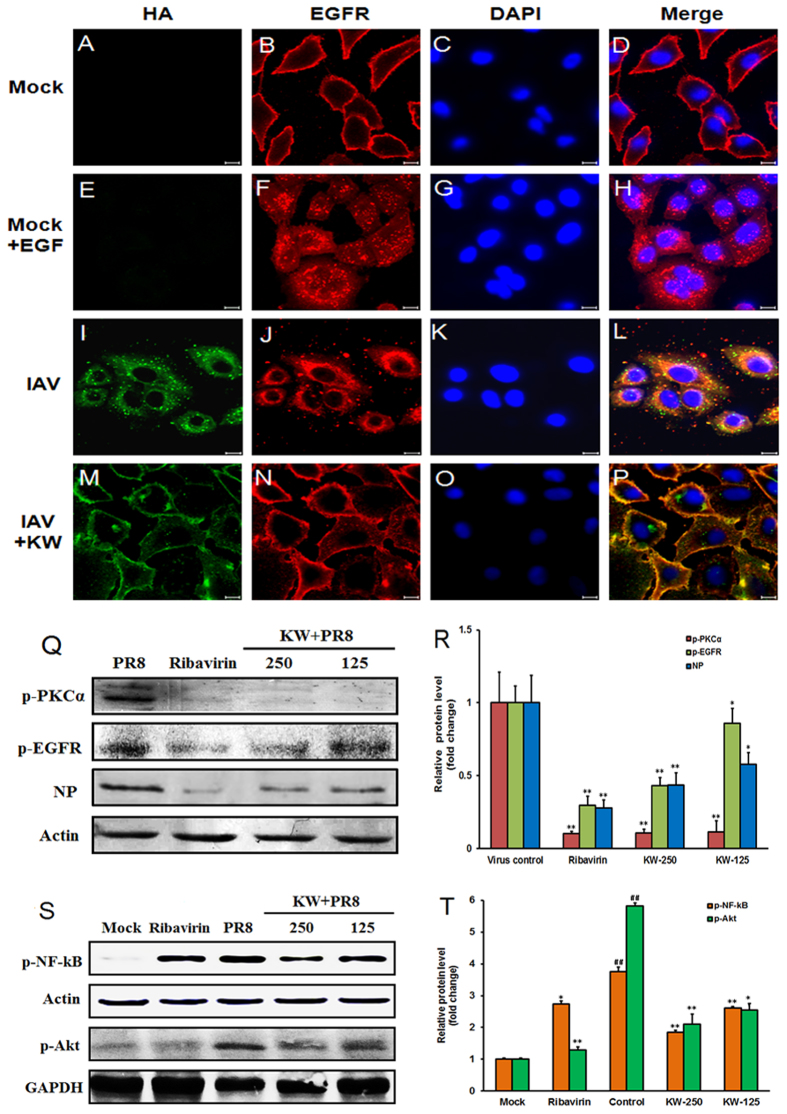
KW reduced IAV endocytosis through inhibition of cellular EGFR pathway. (**A–P**) A549 cells were infected with PR8 virus (MOI = 3.0) with or without KW (250 μg/ml) pretreatment at 37 °C for 1 h, or were stimulated with EGF (100 ng/ml), each for 1 h at 4 °C and 30 min at 37 °C. An EGFR-specific rabbit antiserum and Alexa 594-conjugated goat anti-rabbit IgG as well as a HA-specific mouse antiserum and FITC-conjugated goat anti-mouse IgG were employed. Cells were examined by confocal microscopy. Scale bar represents 10 μm. (**Q**) IAV (MOI = 1.0) infected cells were treated with or without drugs at indicated concentrations after removed virus inoculums. At 4 h p.i., the expression of viral NP protein and phosphorylated PKCα and EGFR proteins were evaluated by western blot. Blots were also probed for β-actin as loading controls. (**R**) Quantification of immunoblot for the ratio of p-PKCα, p-EGFR or NP to β-actin. The ratio for virus control group was assigned values of 1.0 and the data presented as mean ± SD (n = 3). Significance: *p < 0.05, **p < 0.01 vs. virus control group. (**S**) IAV (MOI = 1.0) infected cells were treated with indicated compounds for 4 h, and then the phosphorylation of NF-κB and Akt proteins was evaluated by western blot. Blots were also probed for β-actin and GAPDH as loading controls. (**T**) Quantification of immunoblot for the ratio of p-NF-κB to actin or p-Akt to GAPDH. The ratio for non-infected cells (Mock) was assigned values of 1.0 and the data presented as mean ± S.D. (n = 3). Significance: ^##^*p* < 0.01 vs. normal control group (Mock); *p < 0.05, **p < 0.01 vs. virus control group.

It was reported that some cellular signaling receptors such as epidermal growth factor receptor (EGFR) and fibroblast growth factor receptor (FGFR) are indispensable for IAV entry^8^,^33^,^34^,^35^,^36^, and fucoidans could inhibit the activation of EGFR in some cells^37^. Thus, we then examined whether the inhibition of KW against IAV entry was associated with EGFR pathway by indirect immunofluorescence assay. Briefly, A549 cells were infected with PR8 virus (MOI = 3.0) with or without KW (250 μg/ml) pretreatment at 37 °C for 1 h, or were stimulated with EGF (100 ng/ml), each for 1 h at 4 °C and 30 min at 37 °C. Then the localization of virus HA protein and cellular EGFR protein was detected by immunofluorescence assay. As shown in Fig. 5A–H, EGFR located at the plasma membrane in untreated cells (Fig. 5A–D), while after EGF stimulation, EGFR was internalized into cytoplasm (Fig. 5E–H), which was in concert with the endocytosis process of EGFR reported previously^8^. IAV infection also induced the internalization of IAV particles and EGFR proteins into cytoplasm, and some of them co-localized in the cytoplasm (the yellow dot) (Fig. 5I–L), which was in concert with the previous report that IAV and EGFR were sorted into the same population of late endosomes^8^. However, after KW pretreatment, both IAV particles and EGFR could be rarely detected in the cytoplasm and they mainly located at the cell membrane (Fig. 5M–P), suggesting that the inhibition of KW on IAV endocytosis may be associated with its inhibition of EGFR activation and internalization.

We next explored whether KW could inhibit the activation of EGFR by western blot. As shown in Fig. 5Q,R, after treatment with KW (250, 125 μg/ml) or Ribavirin (50 μg/ml) for 4 h, the levels of phosphorylated EGFR and PKCα protein were significantly reduced compared to that of the non-drug-treated control group (*p* < 0.05), suggesting that KW could inhibit EGFR pathway in IAV infected cells. Moreover, KW also significantly inhibited the expression of viral NP protein at 4 h p.i. (*p* < 0.01), suggesting that KW may block some early stage in IAV life cycle (Fig. 5Q). Thus, KW may block IAV infection through interfering with the activation of EGFR pathway.

To further investigate the inhibition of KW on EGFR pathway, the activation of downstream Akt and NF-κB pathways which is responsible for virus endocytosis and vRNA synthesis was also evaluated. As shown in Fig. 5S,T, the phosphorylated NF-κB significantly increased to 3.8-fold higher than normal control group after IAV infection for 4 h. However, KW (250, 125 μg/ml) treatment significantly inhibited the activation of NF-κB from 3.8 to about 1.8 and 2.6 fold of the normal control group, respectively (*p* < 0.01) (Fig. 5T). Moreover, the activation of Akt protein which is associated with IAV endocytosis was also evaluated (Fig. 5S,T). The results showed that treatment with KW (250, 125 μg/ml) for 4 h significantly decreased the expression level of phosphorylated Akt from 5.8 to about 2.1 and 2.6 fold of the normal control group, respectively (*p* < 0.05) (Fig. 5T). Therefore, the host NF-κB and PI3K/Akt pathways may also be involved in the anti-IAV actions of KW *in vitro*.

#### Intranasal KW application significantly supports survival of mice infected with IAV

**Figure 6:**
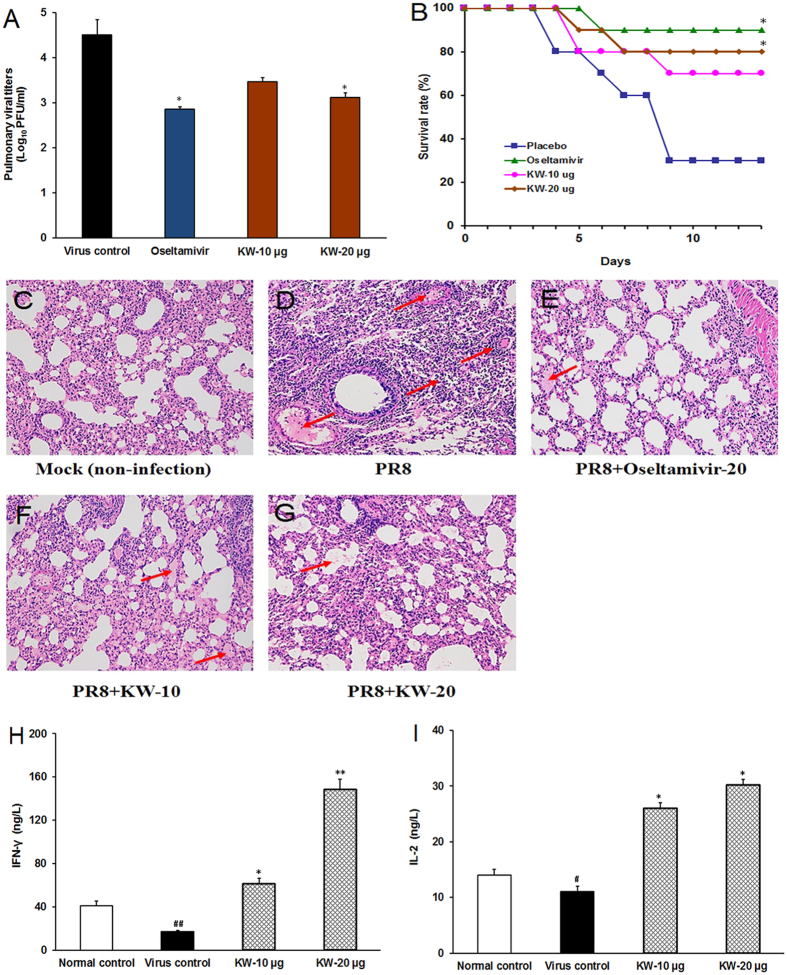
The anti-IAV effects of fucoidan KW *in vivo*. (**A**) Viral titers in lungs. After treatment with KW (10 or 20 μg/day) or placebo (PBS) for 4 days, four mice per group were sacrificed and the pulmonary viral titers were evaluated by plaque assay on MDCK cells. Values are means ± S.D. (n = 3). Significance: *p < 0.05 vs. virus control group. (**B**) Survival rate. IAV infected mice were received intranasal therapy with KW (10 or 20 μg/day) or placebo once daily for seven days. Results are expressed as percentage of survival, evaluated daily for 14 days. Significance: *p < 0.05 vs. control group (placebo). (**C–G**) Histopathologic analyses of lung tissues on Day 4 p.i. by HE staining (×10). The representative micrographs from each group were shown. Mock: non-infected lungs; PR8: IAV infected lungs without drugs; PR8 + Oseltamivir-20: IAV infected lungs with Oseltamivir (20 mg/kg/day) treatment; PR8 + KW-10: IAV infected lungs with KW (10 μg/day) treatment; PR8 + KW-20: IAV infected lungs with KW (20 μg/day) treatment. The red arrows indicate the presence of inflammatory cells in the alveolar walls and serocellular exudates in the lumen. (**H,I**) After treatment of KW (10 or 20 μg/day) for four days, the production of interferon-γ (IFN-γ) (**H**) and interleukin 2 (IL-2) (**I**) in spleen tissues was determined by using the ELISA kits for IFN-γ and IL-2. Values are means ± S.D. (n = 4). Significance: ^#^P < 0.05, ^##^P < 0.01 vs. normal control group; *P < 0.05, **P < 0.01 vs. virus control group.

The anti-IAV effects of fucoidan KW *in vivo* were also explored using a mouse pneumonia model^38^. In brief, IAV-infected mice received intranasal administration of KW (10 and 20 μg/day) or placebo (PBS) once daily for the entire experiment, and the selected subset of treated, infected mice were then sacrificed on Day 4 and the tissue samples were removed for further analysis. Subsequently, the pulmonary viral titers were determined by plaque assay^27^. As shown in Fig. 6A, after treatment of KW (20 μg/day) for four days, the pulmonary viral titers significantly decreased compared to that of the virus control group (*p* < 0.05), suggesting that intranasal therapy with KW could inhibit IAV multiplication in mice lungs. Oseltamivir (20 mg/kg/day) treatment also showed significant reduction of virus titers in mice lungs (*p* < 0.05) (Fig. 6A).

Moreover, the survival experiments were also performed to evaluate the effects of KW on the survival of IAV-infected mice. As shown in Fig. 6B, intranasal administration with KW (20 μg/day) significantly increased survival rates as compared to the placebo-treated control group (*p* < 0.05). By day 14 post infection, only 30% of the individuals in the placebo group survived whereas 80% of animals in the KW (20 μg/day)-treated group survived, comparable to that in Oseltamivir (20 mg/kg/day)-treated group (90%).

To further evaluate the effects of KW on viral pneumonia in mice, histopathology analysis was also performed. As shown in Fig. 6C–G, lung tissues in virus-control group showed marked infiltration of inflammatory cells in the alveolar walls and the presence of massive serocellular exudates in the lumen (Fig. 6D). However, mice treated with KW (10 or 20 μg/day) following infection had intact columnar epithelium in the bronchiole even in the presence of some serocellular exudates in the lumen (Fig. 6F,G). Moreover, the lung tissues with Oseltamivir (20 mg/kg/day) treatment also showed intact columnar epithelium (Fig. 6E). Thus, KW may be able to attenuate pneumonia symptoms in IAV infected mice.

Furthermore, fucoidans were reported to be able to inhibit avian IAV replication through enhancing immune system in mice^19^. Thus, we also explored whether KW could improve antiviral immune system by detecting the production of interferon γ (IFN-γ) and interleukin 2 (IL-2) in IAV infected mice. As shown in Fig. 6H,I, after treatment with KW for four days, the production of IFN-γ and IL-2 in spleens significantly increased as compared to the non-drug treated virus control group (*p* < 0.05), suggesting that the anti-IAV actions of KW *in vivo* may also be associated with its regulation effects on interferon system.

## Discussion

Recently, marine polysaccharides, especially the sulfated polysaccharides derived from marine algae, have attracted increasing interest as potential anti-viral drugs^39^,^40^,^41^,^42^. Fucoidan, a brown algae-derived sulfated polysaccharide, was reported to possess anti-viral activities against different viruses such as HIV and HSV^11^,^12^,^13^,^14^. Herein, fucoidan KW, a high molecular weight sulfated polysaccharide, was demonstrated to be able to suppress the replication of IAV *in vitro* with low toxicity (SI = 80.0). By using single cycle and multi-cycle replication assay, we found that KW could inhibit PR8 (H1N1), Minnesota (H3N2), Cal09 (H1N1) and TX09 (H1N1) virus replication *in vitro*, and the pandemic H1N1 virus (Cal09) was most susceptible to KW treatment (IC_50_ < 6.5 μg/ml). Moreover, compared to the anti-IAV drug amantadine and Oseltamivir, KW had low tendency of induction of viral resistance. Thus, fucoidan KW possesses broad-spectrum anti-IAV activities and low risk of inducing drug resistance.

Fucoidans were reported to have no direct pathogen-killing activity but rather, they inhibit infection *via* blocking virus entry and replication processes^20^. However, in contrast to the previous studies, we found that pretreatment of PR8 or H3N2 virus with KW before infection markedly reduced the viral titers (Fig. 3), suggesting that KW may have direct inactivation effects on IAV particles. By contrast, the addition of KW during virus adsorption or pretreatment of MDCK cells could not significantly decrease the virus titers, which suggested that KW may not interact with MDCK cells directly. Considered that KW did not directly bind HA protein to block virus-induced aggregation of chicken erythrocytes (Fig. 3C), we suppose that pretreatment of IAV with KW before infection may allow KW to fully bind NA and form a stable KW-NA complex to block IAV entry (Figs 4 and 5). Interestingly, post-treatment of cells with KW after adsorption also significantly reduced the virus titers, suggesting that KW may also block some stages after virus adsorption such as entry or release process.

The IAV neuraminidase (NA) protein was reported to be able to promote IAV entry into target cells, in addition to promote the release process of IAV^9^. Herein, we found that KW could induce viral aggregation on the cell surface and inhibit the activity of NA with low IC_50_ values (8.8 μg/ml), suggesting that KW may inhibit IAV infection by interfering with the NA activity of IAV. Moreover, SPR assay showed that KW could bind to the NA proteins of two different subtypes (H1N1 and H3N2) specifically (Fig. 4), suggesting that KW may directly interact with NA to inhibit its activity. Combined with the results that KW could inactivate virus particles directly and block some stages after adsorption (Fig. 3), we hypothesize that KW may inhibit the entry and release process of IAV through direct binding to viral neuraminidase.

IAV entry process often requires engagement of cellular EGFR and FGFR, which are representative receptor tyrosine kinase (RTK) family-members^33^,^34^. The downstream PI3K/Akt and NF-κB signaling of EGFR pathways were reported to be required for efficient virus endocytosis and vRNA synthesis^8^,^35^,^36^. Herein, KW was found to be able to inhibit the phosphorylation of EGFR, PKCα, NF-κB, and Akt proteins in IAV-infected cells (Fig. 5Q–T), which suggested that KW may inhibit the activation of EGFR and its downstream pathways. Considered that KW may hardly cross the cell membrane, we supposed that KW may interfere with the activation of EGFR, thus inhibiting the downstream PI3K/Akt and NF-κB pathways. Moreover, like EGF stimulation, IAV infection could induce the internalization of IAV particles and EGFR into cytoplasm (Fig. 5A–L). However, KW pretreatment could inhibit both IAV endocytosis and EGFR internalization in IAV-infected cells (Fig. 5M–P), suggesting that KW may block IAV endocytosis through interfering with the activation of EGFR. Eierhoff *et al*. proposed a hypothesis that multivalent binding of IAV to sialic acids on the cell membrane can trigger the clustering of RTK monomers, which induces the activation of RTKs to promote the internalization of RTKs and IAV^8^. On that basis, we posit that pretreatment of IAV with KW may allow KW to fully bind to IAV particles to block the multivalent binding of IAV to sialic acids, which interferes with the activation of EGFR pathway, thus inhibiting the invasion of IAV.

The *in vitro* antiviral effects of KW were mirrored in a murine pneumonia model of influenza. Treatment of PR8-infected mice with KW markedly improved their survival and decreased the pulmonary virus titers (Fig. 6). The survival benefits of KW observed in our study may involve a dual mechanism: inhibition of both IAV entry and release process. Moreover, the histopathological analysis indicated that KW treatment could also attenuate the pneumonia symptoms in IAV-infected lungs, which was comparable to the effects of Oseltamivir. Furthermore, KW also enhanced the production of IFN-γ and IL-2 in IAV-infected spleens, suggesting that the anti-IAV actions of KW *in vivo* may also be associated with its regulation effects on the interferon system. Although like other high-molecular weight polysaccharides, KW may hardly cross the different barriers of the body by oral administration, our studies showed that intranasal therapy of KW at low dose (10 or 20 μg/day) had comparable anti-IAV effects to Oseltamivir (20 mg/kg/day) (Fig. 6), which suggested that KW may be used for prevention and treatment of influenza by intranasal administration.

In summary, KW possesses anti-IAV activities *in vitro* and *in vivo*, and may block IAV invasion and release through targeting viral neuraminidase and cellular EGFR pathway. KW possesses broad-spectrum anti-IAV activities and low tendency of induction of viral resistance, superior to the anti-IAV drug amantadine. Further studies of the antiviral effects of KW against highly pathogenic IAV strains (H5N1 or H7N9 strain) will be required to advance it for drug development. In a word, fucoidan KW has the potential to be developed into a novel nasal drop or spray for influenza therapy and prophylaxis in the future.

## Materials and Methods

### Reagents

The fucoidan KW extracted from *Kjellmaniella crassifolia* was provided by School of Medicine and Pharmacy, Ocean University of China. The NA proteins of subtype H1N1 (A/California/04/2009) (11058-VNAHC) and H3N2 (A/Babol/36/2005) (40017-VNAHC) were purchased from Sino Biological Inc. (Beijing, China). FITC-labeled lectins (concanavalin A (ConA), peanut agglutinin (PNA), ulex europaeus I (UEA-I), and wheat germ agglutinin (WGA)) were purchased from Sigma (St. Louis, MO, USA). FITC-labeled maackia amurensis agglutinin I (MAAI) was purchased from Vector Laboratories (Burlingame, CA, USA). Ribavirin (50 mg/mL) was obtained from LuKang Cisen (Jining, China). Oseltamivir carboxylate was purchased from Santa Cruz Biotechnology (Santa Cruz, CA, USA). Oseltamivir phosphate was obtained from Roche (Shanghai, China). Amantadine was purchased from Sigma (USA).

### Cells and virus

MDCK cells were grown in RPM1640 medium supplemented with 10% FBS, 100 U/mL of penicillin and 100 μg/ml of streptomycin. A549 cells were cultivated in F12 medium containing 10% FBS and 2 mM L-glutamine. Influenza virus PR8 was propagated in 10-day-old embryonated eggs for three days at 36.5 °C. Influenza H1N1 virus (A/Texas/15/2009; TX09), (A/California/04/2009; Cal09) and H3N2 virus (A/swine/Minnesota/02719/2009; Minnesota) were propagated in MDCK cells for three days at 37 °C. For infection, virus propagation solution was diluted in PBS containing 0.2% bovine serum albumin (BSA) and was added to cells at the indicated multiplicity of infection (MOI). Virus was allowed to adsorb 60 min at 37 °C. After removing the virus inoculum, cells were maintained in infecting media (RPM1640, 4 μg/ml trypsin) at 37 °C in 5% CO_2_.

### Plaque assay

Confluent cell monolayers in 6 well plates were incubated with 10-fold serial dilutions of IAV at 37 °C for 1 h. The inoculum was removed; cells were washed with PBS and overlaid with maintenance DMEM medium containing 1.5% agarose, 0.02% DEAE-dextran, 1 mM L-glutamine, 0.1 mM non-essential amino acids, 100 U/ml penicillin, 100 μg/ml streptomycin and 1 μg/ml TPCK-treated trypsin. After incubation for 3 days at 37 °C in a humidified atmosphere of 5% CO_2_, cells were fixed with 0.05% glutaraldehyde, followed by staining with 1% crystal violet in 20% ethanol for plaque counting.

### Hemagglutination (HA) assay

The hemagglutination (HA) assay was performed as previously reported^24^,^25^. Standardized chicken red blood cell (cRBC) solutions were prepared according to the WHO manual. Virus propagation solutions were serially diluted 2-fold in round bottomed 96-well plate and 1% cRBCs were then added at an equal volume. After 60 min incubation at 4 °C, RBCs in negative wells sedimented and formed red buttons, whereas positive wells had an opaque appearance with no sedimentation. HA titers are given as hemagglutination units/mL (HAU/mL).

### Cytopathic effect (CPE) inhibition assay

The cytopathic effect (CPE) inhibition assay was performed as described previously^26^,^43^. MDCK cells in 96-well plates were firstly infected with IAV (MOI = 0.1), and then treated with different compounds in triplicate after removal of the virus inoculum. After 48 h incubation, the cells were fixed with 4% formaldehyde for 20 min at room temperature (RT). After removal of the formaldehyde, the cells were stained with 0.1% crystal violet for 30 min. The plates were then washed and dried followed by solubilization of the dye with methanol, and the intensity of crystal violet staining for each well was measured at 570 nm. The concentration required for a test compound to reduce the CPE of IAV by 50% (IC_50_) was determined.

### Time of addition study

MDCK cells were infected with Minnesota or PR8 (MOI = 0.1) under four different treatment conditions: pre-treatment of viruses, pre-treatment of cells, during virus adsorption, or after adsorption. (i) Pretreatment of virus: IAV was pretreated with 250 μg/mL of KW at 37 °C for 1 h before infection. (ii) Pretreatment of cells: MDCK cells were pretreated with 250 μg/mL of KW before infection. (iii) Adsorption: cells were infected in media containing 250 μg/mL of KW and, after 1 h adsorption at 37 °C, were overlaid with compound-free media. (iv) After adsorption: After 1 h adsorption at 37 °C, the inoculum was removed and the infecting media containing 250 μg/mL of KW were added to cells. At 24 h p.i., the antiviral activity was determined by HA assay. Mean percentage HA titers were calculated as a percentage of HA titers from untreated control group.

### Electron microscopy

Confluent cells in 6 well plates were inoculated with PR8 virus for 2 h at 37 °C, and then treated with 250 μg/ml KW or 30 μM Zanamivir in post-adsorption medium for 24 h at 37 °C. Cells were scraped off and centrifuged at 1600 × g for 5 min. Medium was discarded and cells were incubated with ice-cold fixative (2.5% glutaraldehyde in 0.1 M PBS buffer, pH 7.4) for 50 min, with gentle agitation. Cells were pelleted by centrifugation at 20,000 × g for 2 min at RT. Cell pellets were resuspended in 0.5 ml fixative solution then rinsed in 0.5 M cacodylate buffer twice for 10 min and post-fixed with 2% osmium tetroxide for 2 h. The fixed cells were washed with water twice for 10 min, dehydrated with increasing concentrations of ethanol from 50 to 100% and embedded in spurr resin. Thin (70–80 nm) sections were cut on an ultramicrotome and counter stained with uranyl acetate and lead citrate. The sections were viewed and photographed on a JEOL 1010 transmission electron microscope.

### Surface plasmon resonance (SPR) assay

SPR assays were conducted on a SPR biosensor instrument PlexArray® HT C100 (PLEXERA, USA). NA proteins (H1N1 or H3N2) were firstly immobilized onto the surface of a carboxymethylated dextran sensor chip (CM5) *via* amino group coupling as described previously^31^,^32^. To assess real-time binding of KW to the NA proteins on CM5 chips, KW sample with different concentrations (200, 100, 50, 25 nM) dissolved in PBS buffer, was injected over the sensor chip surface with NA immobilized within 3 min, followed by a 5-min wash with PBS buffer. The sensor chip surface was then regenerated by washing with phosphoric acid for 30 s. All binding experiments were carried out at 25 °C with a constant flow rate of 2 μl/s PBS buffer. To correct for non-specific binding and bulk refractive index change, a blank channel without NA was used and run simultaneously for each experiment. Then, the PLEXERA SPR Date Analysis Module (DAM) was used to calculate the kinetic parameters, and the changes in mass due to the binding response were recorded as resonance units (RU).

### Indirect immunofluorescence assay

A549 cells were infected with PR8 virus (MOI = 3.0) with or without KW (250 μg/ml) pretreatment at 37 °C for 1 h, or were stimulated with EGF (100 ng/ml), each for 1 h at 4 °C and 30 min at 37 °C. Then cells were fixed, permeabilized, and incubated respectively with primary antibodies against IAV HA protein or cellular EGFR protein (Santa Cruz, USA) and fluorescein isothiocyanate (FITC)- or Alexa Fluor 594- conjugated secondary antibodies (Boster, Wuhan, China), respectively. Then the cell nucleus was stained with DAPI for 20 min before confocal imaging. Finally, cells were washed and directly observed using Laser Scanning Confocal Microscope (Zeiss LSM 510, Jena, Germany).

### Western blot assay

After drug treatment, the cell lysate was separated by SDS-PAGE and transferred to nitrocellulose membrane. After being blocked in Tris-buffered saline (TBS) containing 0.1% Tween 20 (v/v) and 5% BSA (w/v) at room temperature for 2 h, the membranes were rinsed and incubated at 4 °C overnight with anti-NP protein (Santa Cruz, USA), anti-phosphorylated NF-κB, Akt, EGFR, PKCα antibodies, or anti-β-actin and GAPDH antibodies (Cell Signaling Technology, Danvers, USA) as control. The membranes were washed and incubated with AP-labeled secondary antibody (1:2000 dilutions) at RT for 2 h. The protein bands were then visualized by incubating with the developing solution (p-nitro blue tetrazolium chloride (NBT) and 5-bromo-4-chloro-3-indolyl phosphate toluidine (BCIP)) at RT for 30 min. The relative densities of proteins were all determined by using ImageJ (NIH) v.1.33 u (USA).

### In vivo experiments

Four-week-old female Kunming mice (average weight, 14.0 ± 2.0 g) were housed in polycarbonate cages in a room with controlled humidity and temperature. Fifty mice were randomly divided into five experimental groups (10 mice each). Mice were inoculated intranasally with PR8 (500 PFU/mouse) diluted in 40 μL of 1 × PBS under light anesthesia, and randomly divided into experimental groups. Two hours after inoculation, mice received intranasal therapy of either KW (10 or 20 μg/day), or placebo, and the treatments were repeated once daily for the entire experiment.

Four mice per group were weighed and euthanized on Day 4 after inoculation by spinal dislocation method, and lungs were removed and weighed. The lung specimens of animals from each experimental group were homogenized in 1 × PBS for determination of viral titers by plaque assay. Histopathological analysis was performed using H&E staining on samples collected on Day 4 as described previously^44^.

In the survival experiments, 10 mice per group were intranasally infected with PR8 (1000 PFU/mouse) at Day 0. In addition to the groups with intranasal treatment with KW (10 or 20 μg/day), a group of mice also received an oral dose of oseltamivir phosphate (20 mg/kg/day) as the positive control^45^. The drugs (KW or oseltamivir) administration was repeated once daily for seven days. Mice were monitored daily for weight loss and clinical signs. If a mouse lost body weight over 25% of its pre-infection weight, it was defined as dead and humanely euthanized; the rest of the mice were sacrificed at the end of experiment on 14 dpi.

### Ethics statement

All experiments involving animals were approved by the Institutional Animal Care and Use Committee at Ocean University of China (OUCYY-2016001). All methods were performed in accordance with the animal ethics guidelines of the Chinese National Health and Medical Research Council (NHMRC).

### Statistics

All data are representative of at least three independent experiments. Data are presented as mean ± S.D. Statistical significance was determined using the two-tailed unpaired t-test analysis and the variance analysis (ANOVA). P < 0.05 was considered statistically significant.

## Additional Information

**How to cite this article**: Wang, W. *et al*. Inhibition of Influenza A Virus Infection by Fucoidan Targeting Viral Neuraminidase and Cellular EGFR Pathway. *Sci. Rep.* **7**, 40760; doi: 10.1038/srep40760 (2017).

**Publisher's note:** Springer Nature remains neutral with regard to jurisdictional claims in published maps and institutional affiliations.

## Figures and Tables

**Table 1:** Inhibition effects of different compounds on IAV multiplication *in vitro*.

Compound	Molecular weight (Da)	Sulfate content (%)	IC_50_(μg/ml)	CC_50_(μg/ml)	SI (CC_50_/IC_50_)
KW	536000	30.1	34.4 ± 0.7	2752.6 ± 10.5	80.0
Ribavirin	244	—	24.6 ± 1.5	762.0 ± 8.5	31.0
Oseltamivir	284	—	13.1 ± 1.9	1154.7 ± 18.7	88.1
Amantadine	151	—	23.8 ± 0.6	410.0 ± 7.1	17.2

The inhibition effects on PR8 virus (MOI = 0.1) multiplication in MDCK cells were evaluated by CPE inhibition assay. Inhibition concentration 50% (IC_50_): concentration required to reduce the CPE of the virus by 50% at 48 h p.i. Cytotoxic concentration 50% (CC_50_): concentration required to reduce cell viability by 50%. SI: Selectivity index is defined as the ratio of CC_50_ to IC_50_ (SI = CC_50_/IC_50_).

**Table 2:** Anti-IAV effects of KW over single and multiple cycles of replication.

Subtype	Virus	Single-cycle replication assay^a^		Multicycle replication assay^a^
		HA titer	Infectious virus titer	Plaque number
		IC_50_ (μg/ml)^b^	IC_50_ (μg/ml)^b^	IC_50_ (μg/ml)^b^
H1N1	PR8	44.7 ± 3.7	30.7 ± 2.9	30.5 ± 3.7
H3N2	Minnesota	26.7 ± 2.2	21.4 ± 1.5	6.3 ± 0.1
H1N1	Cal09	6.2 ± 1.4	3.5 ± 0.9	3.8 ± 0.2
H1N1	TX09	5.0 ± 0.7	8.4 ± 1.3	2.9 ± 0.1

^a^Single-cycle high-moi assays and multicycle plaque reduction assays were performed on MDCK cells infected with PR8, Cal09, TX09 and Minnesota. Values are means ± S.D. (n = 3). ^b^Inhibition concentration 50% (IC_50_): concentration required to reduce the virus titer or plaque number by 50% at 48 h p.i.
